# Physiological artifacts in scalp EEG and ear-EEG

**DOI:** 10.1186/s12938-017-0391-2

**Published:** 2017-08-11

**Authors:** Simon L. Kappel, David Looney, Danilo P. Mandic, Preben Kidmose

**Affiliations:** 1 Department of Engineering, Aarhus University, Finlandsgade 22, 8200 Aarhus N, Denmark; 2 Pindrop, 817 West Peachtree Street NW, Suite 770, 24105 Atlanta, GA USA; 3 Department of Electrical and Electronic Engineering, Imperial College, London, SW7 2BT UK

**Keywords:** Ear-EEG, Physiological artifacts, Wearable EEG, Alpha band modulation

## Abstract

**Background:**

A problem inherent to recording EEG is the interference arising from noise and artifacts. While in a laboratory environment, artifacts and interference can, to a large extent, be avoided or controlled, in real-life scenarios this is a challenge. Ear-EEG is a concept where EEG is acquired from electrodes in the ear.

**Methods:**

We present a characterization of physiological artifacts generated in a controlled environment for nine subjects. The influence of the artifacts was quantified in terms of the signal-to-noise ratio (SNR) deterioration of the auditory steady-state response. Alpha band modulation was also studied in an open/closed eyes paradigm.

**Results:**

Artifacts related to jaw muscle contractions were present all over the scalp and in the ear, with the highest SNR deteriorations in the gamma band. The SNR deterioration for jaw artifacts were in general higher in the ear compared to the scalp. Whereas eye-blinking did not influence the SNR in the ear, it was significant for all groups of scalps electrodes in the delta and theta bands. Eye movements resulted in statistical significant SNR deterioration in both frontal, temporal and ear electrodes. Recordings of alpha band modulation showed increased power and coherence of the EEG for ear and scalp electrodes in the closed-eyes periods.

**Conclusions:**

Ear-EEG is a method developed for unobtrusive and discreet recording over long periods of time and in real-life environments. This study investigated the influence of the most important types of physiological artifacts, and demonstrated that spontaneous activity, in terms of alpha band oscillations, could be recorded from the ear-EEG platform. In its present form ear-EEG was more prone to jaw related artifacts and less prone to eye-blinking artifacts compared to state-of-the-art scalp based systems.

## Background

Wearable EEG systems are user-friendly systems enabling long-term recordings in real-life scenarios. Their long-term wearable nature usually comes at the expense of reduced spatial resolution (i.e. fewer electrodes) and less control over interference and artifacts. Most, currently available, wearable EEG systems are too obtrusive and uncomfortable to allow recordings over extended periods of time. However, significant effort has been put into the development of less obtrusive systems [1, 2]. A recent breakthrough is the ear-EEG, where the EEG is measured by electrodes placed on an earpiece inserted into the ear [3, 4]. The ear-EEG methodology supports long-term recordings of EEG in a discreet and comfortable way, without interfering with everyday life activities [5].

Previous studies of visual and auditory evoked potentials in mice have shown that the potentials are significantly different when the mice are moving as compared to not moving [6, 7]. It is likely that similar phenomena would apply to studies in humans. In other words, brain responses observed in natural settings may be different from responses observed under restrained laboratory conditions. Wearable EEG is an enabling technology for this translation of neuroscience from the laboratory to the real-life environment. Thus, the emergence of wearable EEG technology has the potential to open up completely new opportunities in research and medical devices. This include devices for detection of impending hypoglycemic seizures in insulin-treated diabetics [8], monitoring of seizures in childhood absence epilepsy [9], monitoring of driver vigilance [10], brain computer interfaces (BCI) for everyday life communication [11, 12] and neurofeedback to algorithms in hearing aids [13, 14].

An inherent problem when recording EEG is interference arising from noise and artifacts. In a laboratory setting, artifacts and interference can be controlled and to a large extent avoided, but in an uncontrolled real-life scenario this is not possible. Physiological artifacts are a category of artifacts with physiological origin, in contrast to artifacts related to electrical interference. The most significant sources of physiological artifacts are eye blinks, eye movements, and muscle activity [15]. Characterization of physiological artifacts in ear-EEG is particularly interesting, because this category of artifacts cannot be diminished by improving the design of the earpiece or electronic instrumentation, as opposed to artifacts arising from the electrode interface (like e.g. motion artifacts) or electrical interference. Previous studies of artifacts have been performed with scalp EEG and have primarily focused on the characterization of the artifacts [16, 17] and algorithms for automatic detection and removal of artifacts [18, 19].

This paper presents a characterization study of real-life physiological artifacts generated in a controlled environment for nine subjects. In addition, alpha band modulation was studied in an open/closed eyes paradigm. The studies comprised EEG recordings from electrodes distributed over the scalp, and electrodes placed in the ear (ear-EEG).

## Methods

### Quantitative assessment of artifacts

The artifacts were quantified in terms of a signal-to-noise ratio deterioration (SNRD) of a steady-state response (SSR) [20]. The SNRD was calculated as follows. Let *x*(*n*), $n\! =\! 0,\ldots ,N\! -\! 1$, be *N* samples of an EEG signal recorded at a fixed sampling rate under steady-state stimulation. $\omega$ is the normalized angular frequency, [$0,\ldots ,2\pi$], and $X(\omega )$ is the discrete Fourier transform (DFT) of *x*(*n*)

1 $$\begin{aligned} X({\omega })= & {} \sum ^{N-1}_{m=0}{x(n)\cdot e^{-i\cdot \omega \cdot n}}. \end{aligned}$$

The SNR of the SSR for electrode *l* of *L*, $l\! =\! 0,\ldots ,L\! -\! 1$, electrodes can be defined as the ratio between the power of the first harmonic of the SSR and the average power from $\omega _{low}$ to $\omega _{high}$, given by

2 $$\begin{aligned} \mathrm{{SN}}{\mathrm{{R}}_{\;l}} = 10{\log _{10}}\left( {\frac{{|{X_l}({\omega _{SSR}}){|^2}}}{{\,\frac{1}{{{N_{bins}}}}\sum \limits _{\begin{array}{c} \scriptstyle \omega = {\omega _{low}},\\ \scriptstyle \omega \ne {\omega _h} \end{array}}^{{\omega _{high}}} {|{X_l}(\omega ){|^2}} }}} \right) \end{aligned}$$

where $\omega _{SSR}$ is the frequency of the first harmonic of the SSR, $\omega _h$ is the frequencies of the harmonics of the SSR, which are excluded from the noise power estimate, and $N_{bins}$ is the number of included DFT bins from $\omega _{low}$ to $\omega _{high}$. The frequency range defined by $\omega _{low}$ and $\omega _{high}$ do not need to include $\omega _{SSR}$, enabling calculation of the SNR for an arbitrary frequency range.

**Fig. 1:**
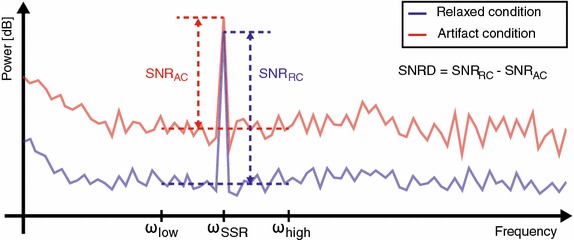
Sketch showing the concept of SNR deterioration (SNRD). The SNR is calculated as the difference between the power of signal and the noise (in dB). The signal is the power at $\omega _{SSR}$ and the noise is the mean power from $\omega _{low}$ to $\omega _{high}$, not including $\omega _{SSR}$. The SNRD is the difference between the SNR in the relaxed condition and the artifact condition in dB

Artifacts were quantified by the SNRD from a relaxed condition to an artifact condition. Let SNR_RC_ be the SNR in a relaxed condition and SNR_AC_ the SNR in an artifact condition. The SNRD is then defined as the difference between SNR_RC_ and SNR_AC_ in dB

3 $$\begin{aligned} \text {SNRD} = \text {SNR}_\text {RC} - \text {SNR}_\text {AC} \end{aligned}$$

as illustrated in Fig. 1.

In the ideal case, the power of the SSR is constant, and the SNRD is the difference between the power (dB) of the noise in an artifact and relaxed condition. However, in practical measurement setups the power of the SSR will vary over time because of changes in e.g. the electrode-skin interface [21]. Assuming that the physiological SSR is constant within a subject for short time windows, the SNRD is not affected by these variations.

### Steady-state stimulus

The artifact study presented in this paper utilized a 40 Hz auditory steady-state response (ASSR) [22]; this paradigm is largely unaffected by attention, cognitive processes, habituation, fatigue, and does not interact with the artifacts under study [23]. Furthermore, as quantifying ear-EEG is a focus of this study, it was natural to choose an auditory paradigm, because previous studies have shown that the ear electrodes are well suited for recording responses from the auditory pathway and primary auditory cortex [3, 4, 20].

The auditory steady-state stimulus was white noise amplitude modulated with 40 Hz. The stimulus was presented to the subjects in both ears by hearing aid speakers (Knowles FK60011) inserted into the ear-EEG earpieces. The stimulus was presented at a sound level well above the hearing threshold.

### Experimental setup

EEG were recorded in a controlled laboratory setting, where the subjects were seated in a comfortable chair. The EEG was acquired with three synchronized 16 channel g.USBamp EEG amplifiers (g.tec, Austria). Two amplifiers were used to record scalp EEG and one amplifier was used to record ear-EEG.

The scalp EEG were recorded from 32 active g.LADYbird electrodes (g.tec, Austria). The scalp electrodes were located according to the 10–20 system at positions F8, FC4, FC6, FT8, C2, C4, C6, T8, CP4, CP6, TP8, TP10, P4, P6, P8, Fz, FCz, F7, FC3, FC5, FT7, C1, C3, C5, T7, CP3, CP5, TP7, TP9, P3, P5, and P7. All scalp electrodes were referenced to the Cz electrode and the GND electrode was placed on the left cheek. A Cz reference is convenient for characterization of physiological artifact, because the tissue below the Cz electrode does not contain any muscles. Thus, artifacts from the electrical activity in muscles will be limited at this location.

**Fig. 2:**
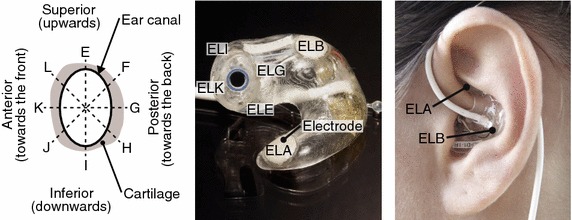
Ear-EEG earpieces with electrode labels. *Left* ear-EEG labeling convention for the ear-canal electrodes, when looking into the left ear-canal. *Middle* picture of a left earpiece with indication of the electrode labels. *Right* the ear-EEG earpiece inserted in the left ear. *Labels* indicate the positions of the ELA and ELB electrodes in the concha part of the outer ear

The ear-EEG were recorded from passive silver electrodes embedded on the surface of custom made earpieces as described by Looney et al. [5]. The ear-EEG electrode label convention were Exy, where x denotes the left (L) or right (R) ear, and y the position within the ear. Two electrodes were positioned in the concha part of the ear and labeled ExA and ExB. In addition, four electrodes were located in the ear-canal and labeled ExE, ExG, ExI, and ExK as shown in Fig. 2. The labeling convention was defined by Kidmose et al. [3].

The ear electrodes were referenced to the ExB electrodes and the ExA electrodes were connected to the amplifier’s GND. The ExB electrodes were chosen as references to optimize the inter-electrode distance and ASSR. A previous study of reference configurations for ear-EEG, showed that a reference electrode located in the concha part of the ear is a good choice for recording the ASSR with ear-EEG [24]. The left earpiece, the scalp, and the right earpiece were connected to galvanic isolated groups on the EEG amplifiers, and had different reference and GND electrodes as described above. Prior to insertion of the earpieces, the ears were cleaned with alcohol and skin preparation gel (Nuprep Skin Prep Gel). A high viscosity conductive gel (Elefix EEG paste) was applied to the ear electrodes before insertion. 9 subjects (7 males) with no history of neurological disorders and normal audiological status aged between 24 and 42 (mean = 29 ± 5) years, participated in the study.

### Processing of EEG for artifact quantification

The EEG recordings, for the quantitative assessment of artifacts, were preprocessed with a Hamming windowed sinc finite impulse response (FIR) bandpass filter with an order of 9901 and cutoff frequencies of 0.2 and 120 Hz. The filter was implemented with the EEGLAB function “pop_eegfiltnew” [25]. In addition, a 50 Hz and a 100 Hz second order IIR notch filter were applied to the EEG data to attenuate the first and second harmonics of power line interference in the measured EEG.

100 s of data from a relaxed condition and 100 s of data from an artifact condition were extracted from each recording, and SNR values were calculated as described by Eq. (2) for different configurations of $\omega _{low}$ and $\omega _{high}$. A data sequence, recorded by an electrode, was discarded if the first harmonic SSR in the relaxed condition was not statistically significant (p > 0.05) different from the mean noise in the interval from $\omega _{low}=32$ Hz to $\omega _{high}=48$ Hz, measured by an F test [26]. The F test is commonly used in ASSR-based hearing threshold estimation [26–28]. The discarding was performed to ensure that the SNRD were reliable for all included data sequences.

The experiment was divided into 4 min recordings. Each recording contained 2 min where the subject was generating artifacts, and for the remaining 2 min the subject where in a relaxed condition. ASSR stimulation was performed during all 4 minutes. This enabled calculation of both the SNR_RC_ and SNR_AC_ for each recording, resulting in a more robust estimate of the SNRD, calculated as described by Eq. (3).

### Artifact conditions

A paradigm comprising four groups of artifacts was designed. Each group included different artifact conditions, with each condition designed to be reproducible and mimic a common real-life EEG artifact.

#### Jaw artifacts

The characterization of jaw-related artifacts was divided into three artifact conditions

- *Jaw clenching* The subjects were instructed to clench their teeth with maximum strength for intervals of 30 s.
- *Controlled jaw move* A custom-made device was used to ensure a continuous and repeatable movement of the jaw; the device is described in [20]. The subjects were instructed to bite around the tip of the device and follow its movement, causing a controlled, repeated movement of the jaw from 3 to 12 mm teeth-to-teeth opening with a period of 3 s. Jaw movements were created for intervals of 30 s. This method created jaw movements with only limited muscle activity.
- *Biting* The subjects were instructed to bite around the tip of the custom-made device and hold the bite with maximal force for intervals of 30 s. The condition imitated biting around e.g. food, and is a more realistic everyday life condition, compared to the jaw clenching condition.

#### Eye-blinking

In the eye-blinking artifact condition the subjects fixed their gaze on a dot in the center of a cross displayed on a monitor. The subjects were instructed to perform an eye-blink whenever the cross flashed. The cross flashed every second for intervals of 30 s. To prepare the subject for a flashing cross, the dot in the center gradually decreased in size until the cross flashed.

The monitor was a 32” LCD display ($569\times 343$ mm^2^) placed in front of the subject at a distance of 600 mm from the subject’s forehead. A chin-rest was used to keep the head steady during the recordings.

#### Eye movement

For the eye movement artifact conditions, the subjects were instructed to fix their gaze on a ball (diameter of 7 mm) displayed on the monitor. The ball had two states; in motion and steady. In motion the ball moved horizontally or vertically with a period of 4 s and followed a sinusoidal function, causing the position, velocity, and acceleration of the eyes to become continuous. When the ball was steady it was positioned in the center of the monitor. The state was changed every 30 s. A chin-rest was used to keep the head steady during the recordings. The motion of the ball corresponded to a ±25^o^ and ±16^o^ horizontal and vertical movement of the eyes, respectively.

#### Head movement

For the head movement artifact conditions, a ball, displayed on the monitor, moved with the same patterns as described previously for the eye movement. The subjects were instructed to follow the motion of the ball by rotating their neck. To reduce eye movements, the subjects wore goggles with a restricted field of view. The glass of the goggles was covered with a frosted window foil, and the field of view was limited to a single hole in the center of the goggles.

### Alpha band modulation

The quantitative assessment of artifacts, presented above, was based on the ASSR. In order to investigate the quality of spontaneous EEG recorded with ear-EEG, alpha band modulation was studied in an open/closed eyes paradigm. Similar recordings were presented in [4]. The current study differs in that the ear-EEG was recorded with both the reference and the GND connected to ear electrodes, thus corresponding to a situation where the ear-EEG is used as a standalone device. In addition, we present both power and coherence measures.

Before the recordings, the subjects were instructed for two conditions: (1) simple arithmetic task with open eyes; (2) relaxing with closed eyes. An auditory cue indicated a change in condition every 60 s. The first condition was always condition 1. The simple arithmetic task was to repeatably subtract 7 from a random number in the interval between 50 and 200. A new number was given every 10 s.

The recordings were bandpass filtered with a Hamming windowed sinc FIR filter with an order of 1981 and cutoff frequencies of 1 and 46 Hz. The filter was implemented with the EEGLAB function “pop_eegfiltnew” [25]. Power spectrograms were calculated with a segment size of 4 s and an overlap between segments of 3 s. The same segments were used for calculation of the magnitude squared coherence. The coherence was calculated for each segment and was based on Welch method to estimate the cross and auto spectra; hence for the calculation, each segment was divided into 20 subsegments each with a size of 2 s and an overlap of 1.9 s.

The grand average alpha power and coherence were also calculated for each segment. Initially the total alpha (8–12 Hz) power and the mean alpha coherence were calculated for each segment. For each recording, the mean alpha power of all segments were subtracted from the alpha power of each segment. The grand average alpha power was then calculated for each segment. The grand average alpha coherence was calculated similarly, but without subtracting the mean alpha coherence from each segment. The temporal course of the grand average alpha power and coherence were smoothed with a 3-tap mean filter.

## Results

### Quantitative assessment of artifacts

Data from the 9 subjects, 9 conditions, and 32 scalp and 8 ear electrodes were processed, resulting in 648 ear and 2592 scalp data sequences. Based on the rejection criteria described in the “Processing of EEG for artifact quantification” section, a total of 22 % (140/648) of the ear-EEG and 26 % (668/2592) of the scalp EEG data sequences were discarded. For the subject with the most rejected EEG data, 46 % (164/360) of the data sequences were discarded.

**Fig. 3:**
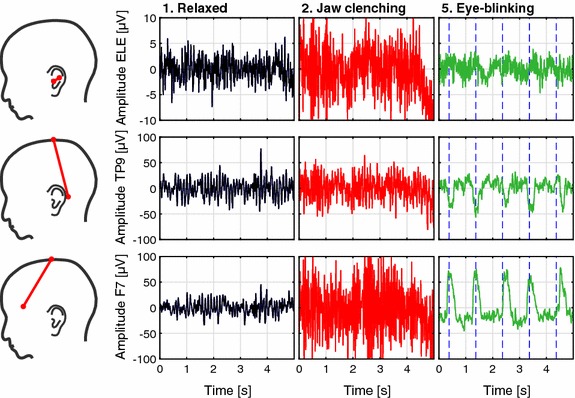
Time domain examples of recordings from a single subject. The *first row* shows recordings from the ELE-ELB electrode pair. The *second* and *third row* are recordings from the TP9-Cz and F7-Cz electrode pairs, respectively. The *sketches* on the *left* illustrate the electrode positions. The *plots* show raw EEG data band-pass filtered from 1 to 40 Hz. The *blue dashed lines* for the eye-blinking condition indicate the eye-blink cue

Figure 3 shows typical time domain examples of EEG recordings from the relaxed, jaw clenching and eye-blinking conditions for a single subject. The first row in Fig. 3 shows recordings from the ELE-ELB electrode pair, the second and third row are recordings from the TP9-Cz and F7-Cz electrode pairs, respectively. The plots show an approximately 20 dB lower amplitude for ear-EEG compared to scalp EEG, but a proportionally comparable increase in the noise level from the relaxed to jaw clenching condition. Eye-blinking is clearly visible in recordings from the scalp electrodes, and not immediately visible in the ear-EEG recording.

**Fig. 4:**
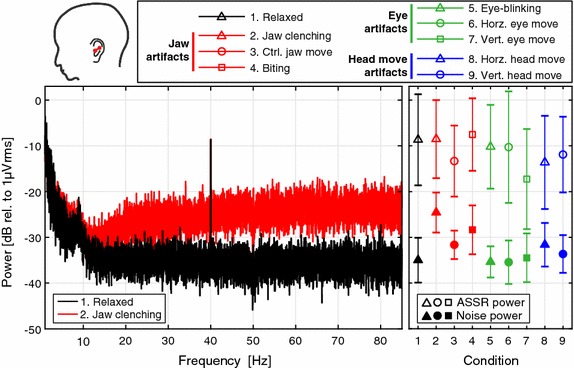
Grand average power spectra for the ELE ear electrode. *Left* grand average power spectra for the relaxed and jaw clenching condition from the ELE–ELB electrode pair, as indicated by the *red line* sketched on the head drawing in the *top*. *Right* the *markers* indicate the grand average ASSR power and mean noise power from 32 to 48 Hz. The *error bars* denote ±1 standard deviation, calculated as the grand standard deviation of the ASSR or noise power in dB

**Fig. 5:**
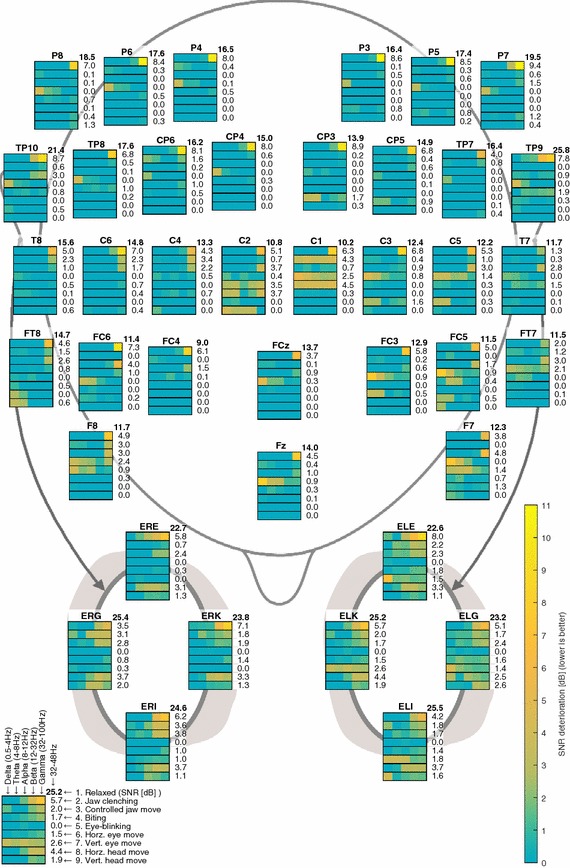
Overview of SNRD values for the studied artifact conditions. The SNRD values are expressed as a *color-code* for the clinical frequency bands, delta (0.5–4 Hz), theta (4–8 Hz), alpha (8–12 Hz), beta (12–32 Hz) and gamma (32–100 Hz). In addition, the SNRD is given in numbers for the frequency band from 32 to 48 Hz. For the relaxed condition, the SNR is given instead of the SNRD value. Negative SNRD values were set to 0 dB

**Fig. 6:**
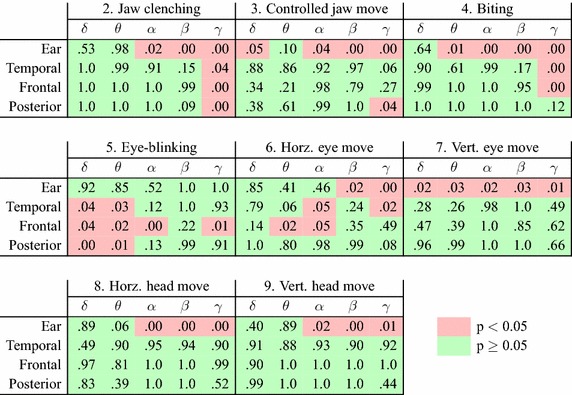
Tables of p values for t tests of the statistical significance of the SNRD values. The tables show p values for different frequency bands, electrode groups, and artifact conditions. Each of the tests was a paired one-sided t test of difference in the mean SNR power ratio for a relaxed and artifact condition. The electrodes were arranged in groups, containing the following electrodes: ear = {ELE, ELG, ELI, ELK, ERE, ERG, ERI, ERK}, temporal = {TP7, TP9, C5, T7, FT7, TP8, TP10, C6, T8, FT8}, frontal = {F7, FC3, FC5, FCz, Fz, F8, FC4, FC6}, occipital = {CP3, CP5, P3, P5, P7, CP4, CP6, P4, P6, P8}

The results in Figs. 4, 5 and 6 are presented with a Cz reference for all scalp electrodes, an ELB reference for left ear electrodes and an ERB reference for right ear electrodes. The left panel in Fig. 4 shows grand average power spectra for the relaxed and jaw clenching condition from the ELE electrode. A clear ASSR is observable at 40 Hz for both conditions, and differences in the noise level can be observed; e.g. for jaw clenching the noise is increasing from 10 Hz with a plateau from 60 to 80 Hz, where the noise level is highest. The right panel shows statistics for all conditions in terms of the grand average and standard deviation of the ASSR power and mean noise power from 32 to 48 Hz. The right panel display a relatively high inter-subject variability of the ASSR power, compared to the inter-subject variability of the noise power. It can also be observed that the noise power is highest for the jaw clenching and biting conditions.

Figure 5 provides an overview of the SNRD values for the artifact conditions measured in various electrode locations and frequency bands. Figure 6 shows tables of p values for paired one-sided t tests of the statistical significance of the SNRD values for different frequency bands, electrode groups, and artifact conditions. Using the electrode groups from Fig. 6, the data analysis showed that the SNR for the ear electrode group were significantly higher than the SNR for the scalp electrode groups in the relaxed condition. The p values for two-sample t tests of difference in the mean SNR values were statistical significant (p < 0.001) for all ear to scalp electrode groups (SNR_ear_ = 24dB, SNR_temporal_ = 19dB, SNR_frontal_ = 12dB, SNR_posterior_ = 17dB).

The SNRD values in Fig. 5 show that jaw clenching and biting artifacts were present all over the scalp and ears, with the highest SNRD values in the gamma band (32–100 Hz). This is supported by Fig. 6 which show that the SNRD values were statistical significant (p < 0.05) in the gamma band across all electrode groups for jaw clenching, and for the ear, temporal and frontal electrode groups for biting. For the ear electrodes, jaw clenching and biting artifacts were also statistical significant in the alpha (8–12 Hz) and beta bands (12–32 Hz). Jaw movement artifacts were mainly statistical significant for the ear electrodes, where the SNRD values were statistical significant for the delta, alpha, beta and gamma bands. In general, jaw artifacts were higher for the ear electrodes, compared to the scalp electrodes.

Artifacts from eye-blinking were not statistical significant for the ear electrodes. However, for the scalp electrodes the SNRD were statistical significant in the delta (0.5–4 Hz) and theta (4–8 Hz) bands and most pronounced in frontal electrodes.

Horizontal eye movements mainly affected the frontal electrodes in the theta and alpha bands and temporal electrodes in the alpha band. Statistical significant values were also measured in the gamma band for the temporal electrodes and in the beta and gamma bands for ear electrodes. For the vertical eye movement condition, statistical significant artifacts were measured from the ear electrodes in all the investigated frequency bands, whereas no statistical significant artifacts were measured from scalp electrodes.

Artifacts from head movements were only statistical significant for the ear electrode group in the alpha, beta and gamma bands.

### Alpha band modulation

**Fig. 7:**
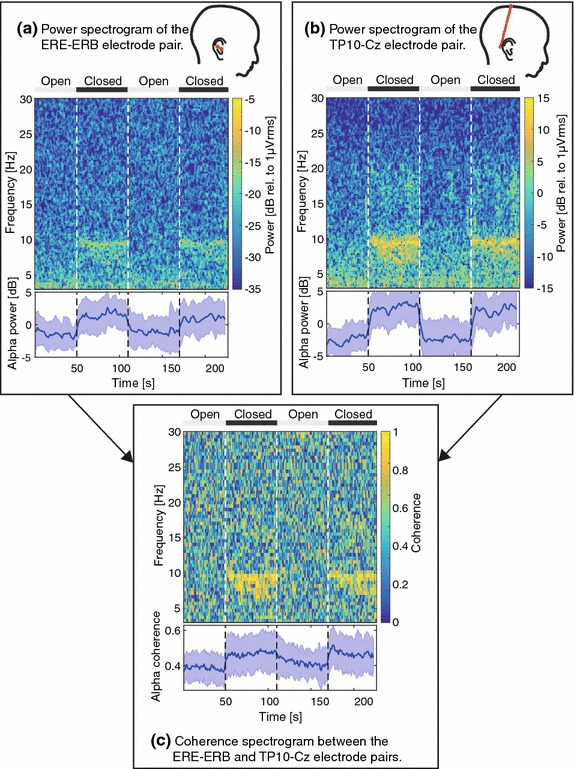
Spectrograms of the alpha band modulation recordings. Each subplot shows a spectrogram for one subject with the grand average alpha power or coherence plotted below. **a** Power spectrogram of the ERE-ERB electrode pair. **b** Power spectrogram of the TP10-Cz electrode pair. **c** Spectrogram of the coherence between the ERE-ERB and TP10-Cz electrode pairs. The *white dashed lines* denote the instances of changes between open and closed eyes. The *shaded area* of the grand average plots is the standard deviations of the grand averages

Figure 7 shows power and coherence spectrograms for the ERE-ERB and TP10-Cz electrode pairs for one subject. The grand average alpha power and coherence are plotted below the spectrograms. The open and closed eyes intervals are clearly distinguishable by increased alpha power and coherence during closed eyes.

## Discussion

### Quantitative assessment of artifacts

The significantly higher SNR of the ASSR for the ear-EEG compared to scalp EEG is consistent with previous ASSR recordings performed with ear-EEG [3, 20]. Figures 3, 4, and 5 generally show the highest artifact level for the jaw clenching and biting conditions. Previous studies conclude that the main contributor to jaw clenching and biting artifacts is electromyography (EMG) related to increased tension in the jaw muscles during biting [17, 29]. Figure 6 shows that the SNRD for the jaw clenching and biting conditions were statistical significant in the gamma band for the ear, temporal, and frontal electrode groups, corresponding well with the assumption of EMG as the dominant artifact source.

For the controlled jaw move condition, the subjects were asked to relax their jaw muscles during the experiment, enabling an investigation of artifacts related to jaw movement with a minimal EMG contamination of the EEG. For the ear electrodes, the artifacts were statistical significant for the delta, alpha, beta and gamma bands. The artifacts in the delta band were likely motion artifacts related to changes in the shape of the ear-canal, caused by the jaw movements [30–32]. Some of the motion artifacts associated with jaw movements might be reduced by constructing the earpieces in a soft material, which would allow the earpiece to adapt to changes in the shape of the ear-canal. Studies of jaw movements have shown, that movements of the ear-canal relative to the concha part of the ear, cannot be described by deformation of the ear-canal alone, thus, the concha is also deformed during jaw movement [31, 32]. Based on this observation, it would be beneficial to mechanically decouple the concha and ear-canal part of the earpiece. This could be obtained by a flexible joint between the concha and ear-canal part, or by dividing the earpiece in to two separate components. A secondary aspect of the artifacts related to jaw movements could be related to skin-stretching. Previous studies have shown that stretching of the skin changes the potential over the epidermis, which could affect both scalp and ear electrodes [33].

Eye-blinking artifacts were only statistical significant for scalp electrodes, and mainly in the delta and theta bands, but also in the alpha and gamma bands for frontal electrodes. This is in line with previous studies of eye-blinking [34, 35]. Eye-blinking artifacts were not statistical significant for ear-EEG recordings, corresponding well with the general experience of eye-blinking artifacts in a large number of ear-EEG recordings performed in our lab. The EEG artifacts related to eye-blinking are thought to primarily originate from the eyelid functioning as a conductor between the corneal surface and the fronto-polar region of the scalp, creating a positive potential in the frontal electrodes during an eye-blink [34, 36].

The statistical significant artifacts observed in theta and alpha bands for frontal and temporal scalp electrodes during horizontal eye move were probably electrooculography (EOG). EOG is related to the movement of the eyeball, which is electrically polarized; positive at the cornea and negative at the retina [37]. Thus, the origin of the artifacts observed for the eye-blink and eye movement conditions were most likely not the same. Previous studies have reported artifacts related to both vertical and horizontal eye movement [34, 35], and it is unclear why the SNRD for vertical eye movement were not statistical significant for scalp electrodes in the current study. Statistical significant SNRD values were also observed in the gamma bands for the eye movements artifacts. We speculate that this could be related to tension in the jaw muscles. As ear electrodes are more prone to jaw artifacts than scalp electrodes, the SNRD in the beta and gamma bands during eye movements may be due to the chin-rest, rather than the eye-movements as such.

Vertical and horizontal head movements only caused statistical significant SNRD values for ear electrodes in the alpha, beta and gamma bands. The scalp EEG was measured with active electrodes, and the ear-EEG was measured with passive electrodes. Based on this difference in electrode technology, it is likely that the ear-EEG recordings were more affected by capacitive coupled noise and noise related to cable motions. Thus, the artifacts, observed in the ear, could originate from cable motions rather than head movements as such. Cable motions can be reduced by mounting the EEG amplifier on the head, as demonstrated by Debener et al. [2].

In order to enable inclusion of eight artifact conditions in the study, within an acceptable time frame, each artifact condition was exercised for only 2 min. For some subjects, 2 min were not long enough to obtain a statistical significant ASSR for all electrodes, causing relatively high percentages of discarded data in the study, as reported in the “Results” section.

### Alpha band modulation

The recordings of alpha band modulation showed that spontaneous EEG can be recorded with the described ear-EEG setup, where the measuring, reference and GND electrodes were located within the same ear. The increased alpha coherence during closed-eyes periods indicates a common source of the alpha oscillations observed for the ERE-ERB and TP10-Cz electrode pairs, as shown in Fig. 7. The observations correspond well with previous studies of alpha band modulation performed with scalp EEG [38, 39], and ear-EEG [4, 5].

## Conclusions

Methods for assessing the interference from artifacts were developed, and the methods were applied to recordings from 8 artifact conditions and 9 subjects. Analysis of the artifacts was based on the auditory steady-state response (ASSR) and artifacts were quantified through the signal-to-noise ratio deterioration (SNRD) of the first harmonic of the ASSR.

Jaw clenching and biting were the most severe artifacts in both scalp and ear electrodes. Jaw movement artifacts were mainly statistical significant for ear-EEG, and likely related to changes in the shape of the ear-canal. Artifacts related to eye blinking were only statistical significant for scalp electrodes, and were highest for frontal electrodes. Eye movements created statistical significant artifacts in frontal, temporal, and ear electrodes. The study confirmed previous observations of a statistical significant higher SNR of the ASSR for ear-EEG compared to scalp EEG. In addition, alpha band modulation were studied in an open/closed eyes paradigm, where increased power and coherence were observed in the alpha band for ear and scalp electrodes during the closed eyes intervals.

Generally, the results from the quantitative study of artifact and recordings of spontaneous EEG are promising for the future development and application of the ear-EEG technology in discreet, unobtrusive and user-friendly devices for recording of EEG in real-life settings.
